# Rectal dosimetry in intracavitary brachytherapy by HDR at rural center of Maharashtra: Comparison of two methods

**DOI:** 10.4103/0971-6203.51936

**Published:** 2009

**Authors:** Rajeev Shrivastava, Rahul B. Umbarkar, M. B. Sarje, K. K. Singh

**Affiliations:** Department of Radiotherapy and Oncology, Rural Medical College, Loni, Ahmednagar, Maharashtra, India

**Keywords:** International Commission on Radiation Units and Measurements (ICRU 38), Intracavitary radiotherapy, rectal dose

## Abstract

The purpose of this study was to calculate the radiation dose at the anterior rectal wall as per the International Commission on Radiation Units and Measurements (ICRU 38) recommendations and compare it with the dose calculated by the commonly used intrarectal catheter. Dose delivery by brachytherapy to the cervix is limited by the critical structure of the bladder and rectum. In this study the ICRU-38 rectal point was derived by using a radio-opaque gauze piece on the posterior vaginal wall, and the intrarectal point was derived by inserting a rubber catheter with a wire, inside the rectum. A total of 146 applications were performed in 81 patients. Rectal doses were compared for complementary rectal points R1 and R5, R2 and R6, R3 and R7, and R4 and R8, obtained by both methods. The rectal doses at each complementary pair were compared with each other. The average dose at R1 was 5% higher than at R5 (60.57% vs. 55.57%). The average dose at R2 was 1% higher than at R6 (58% vs. 57%). The average dose at R3 was 1.29% higher than at R7 (52.71% vs. 51.42%), and the average dose at R4 was 1.15% higher than at R8 (43% vs. 41.85%). There were many instances where the rectal dose exceeded by more than 15%, from the R1 to R4 points (43, 22, 21, and 11 times, respectively, for R1-R5, R2-R6, R3-R7, and R4-R8 pairs). The difference in dose between R1 and R5 was significant as seen on the statistical tests, i.e., Pair T test, Wilcoxan Signed Ranks test, and Sign test (p value 0.002). The rectal dose obtained by the intrarectal wire method underestimates the actual dose to the rectum when compared to the ICRU-38 method. Thus ICRU-38 recommendations should be strictly adhered to, to reduce late complications.

## Introduction

The combination of external beam radiotherapy (EBRT) and intracavitary brachytherapy (ICRT) has been well established in the definitive management of cervical cancer. The bladder and rectum are two organs that act as dose limiting organs, due to their low tolerance. The most important treatment-related factors that that could lead to a creation of late complications on the rectum include, total dose to the rectum, volume of the irradiated rectum, and dose rate of the brachytherapy modality used.[1–4] If a higher dose is delivered to these critical organs, it leads to late complications, with a decrease in the quality of the patient's life.[5] To overcome this problem brachytherapy is added, which delivers higher doses to localized regions of the cervix and a lesser dose to the bladder and rectum primarily, as per inverse square law, and also due to absorption and scatter in the intervening media. The International Commission on Radiation Units and Measurements (ICRU 38) recommendss[6] certain guidelines for measurements and reporting of intracavitary insertions, as it may vary from center to center. As per ICRU-38, the posterior vaginal wall is visualized by means of an intravaginal mould or radio-opaque gauze. The rectal reference point is determined on a lateral radiograph, on the anteroposterior (AP) line drawn through either the lower end of the intrauterine source or through the middle of the intravaginal sources, 5 mm behind the posterior vaginal wall. Many centers use the intrarectal catheter to visualize the rectum, and a point 5 mm anterior to this is taken as the rectal point, for calculation. The calculation of the rectal dose is very important as a higher rectal dose often leads to increased morbidity in patients, even in successfully treated patients.

This study was undertaken to estimate and compare the rectal dose by intrarectal catheter and ICRU reference point.

## Materials and Methods

**Figure 1:**
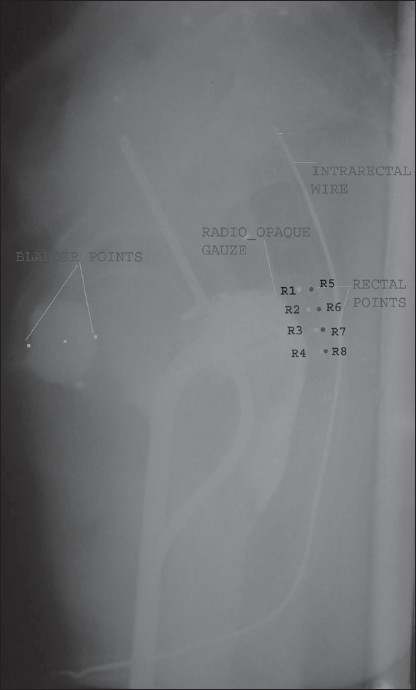
Lateral film with ICRU-38 as well as intrarectal points marked

This study was undertaken in a prospective way from October 2006 to January 2008. Eighty-one patients of proven carcinoma cervix, stage IIB to IIIB, were taken for the study. All the patients received EBRT at the dose of 50 Gy/25#/5 weeks. All the patients were planned for three to four fractions of ICRT via the High Dose Rate Brachytherapy machine (HDR), as per the stage. During each application, an intrauterine tandem (4-6 cm) was placed into the uterine cavity, with ovoids (1.5-2.5 cm) in the vagina, at the level of the fornices. A radio-opaque gauze (barium soaked) was placed on the posterior vagina followed by proper packing with a povidine iodine-soaked gauze piece to further displace the bladder anteriorly and rectum posteriorly. A rectum marker, using a radio-opaque metallic wire inside the hollow rubber catheter of 1 cm diameter, was also placed inside the rectum. Orthogonal films were taken and rectal points were marked on the lateral x-ray film as R1-R4, 0.5 cm behind the posterior most visualized portion of the barium-soaked gauge, with R1 at the level of the cervical os, i.e., on the lower end of intrauterine source. Similarly points R5-R8 were marked 0.5 cm anterior to the rectal catheter with R5 at the level of the cervical os [Figure 1]. The distance between each point in both sets was taken as 1 cm. The points were selected symmetrically in relation to the anteroposterior line passing through the middle of the intravaginal sources. In all insertions a dose of 7 Gy was given at point A. Planning and dose distribution were calculated using the Abacus treatment planning software for each complementary rectal point, i.e., R1 and R5, R2 and R6, R3 and R7, and R4 and R8.

### Statistics

For all rectal points, mean, median, maximum, and minimum doses were calculated. For the R1 and R5 pair, Paired T test, Wilcoxan Signed Ranks test, and Sign test were performed using SPSS statistical software version 10.0, for assessment of significance.

## Results

There were a total of 146 insertions of brachytherapy. The mean, median, maximum, and minimum doses for rectal points R1-R8 are shown in Table 1.

**Table 1:** Rectal doses at complimentary points by the two methods

*Rectal doses (Gy)*	*Points by ICRU method*				*Points by rectal wire method*			

	*R1*	*R2*	*R3*	*R4*	*R5*	*R6*	*R7*	*R8*
Mean	4.24	4.06	3.69	3.01	3.89	3.99	3.60	2.93
Median	4.19	4.06	3.65	2.92	3.96	3.99	3.42	2.78
Maximum	11.13	8.75	8.3	5.89	7.62	6.89	7.67	6.46
Minimum	1.20	1.03	0.87	0.76	0.94	091	0.82	0.74

The average dose at R1 was 5% higher than at R5 (60.57% vs. 55.57%). The average dose at R2 was 1% higher than at R6 (58% vs. 57%). The average dose at R3 was 1.29% higher than at R7 (52.71% vs. 51.42%). The average dose at R4 was 1.15% higher than at R8 (43% vs. 41.85%). There were many instances where the rectal dose was exceeded by more than 15% at the R1 to R4 points (43, 22, 21, and 11 times respectively for the R1-R5, R2-R6, R3-R7, and R4-R8 pairs).

**Figure 2:**
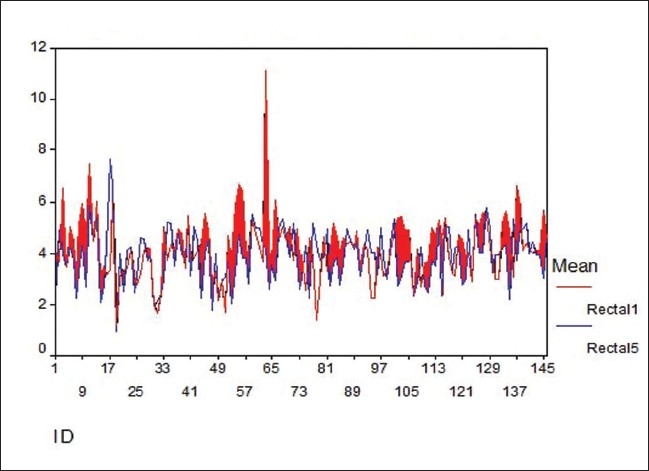
Comparison of the mean of rectal doses at R1 and R2

The pair T test was applied to R1 and R5, R2 and R6, R3 and R7, and R4 and R8 pairs. For the R1 and R5 pair, the mean were 4.24 and 3.88 and the standard deviation 1.23 and 1.06, respectively. The 95% confidence interval of the difference was between 0.1341 and 0.5763 with a t value 3.175 (p value 0.002), which was significant. For the other three pairs there was no significant difference. On applying the Wilcoxan Signed Ranks test, on 87 occasions R1 was greater than R5 (Sum of ranks = 6766), whereas, on 57 occasion R5 exceeded R1 (Sum of Ranks = 3674). Thus the Z value for the test was −3.083 (p value 0.002), which was significant. Similarly, the Sign test also gave the value of Z as −2.417 (p value 0.016) for the R1 and R5 pair, which is again significant. For the other three pairs these tests were not significant. The doses of R1 and R5 were plotted against their mean [Figure 2]. It shows that the dose at R1 is significantly higher than that at R5.

## Discussion

In the recent era, the dosimetry for ICRT has seen many changes, with the computed tomography scan (CT scan) being increasingly used for planning.[7] However, these facilities are limited to only a few urban centers. Rural centers in particular lack these facilities. Still most of the centers use the traditional method of orthogonal film. These films are used for dosimetry of ICRT as per ICRU-38 recommendations. Doses can be calculated for Manchester points A, B, rectal, and bladder reference points. Spatial orientation of the applicator, with relation to fixed bony points, can be seen by these orthogonal films. Specification of the region of rectal mucosa that absorbs the highest doses, as per ICRU-38 recommendations, is valid, and serves as a means of comparison among radiotherapy centers. These rules should be obeyed by all the centers to have uniformity in reporting the cases. To calculate the rectal dose many centers use flexible wire markers inside the rectum.[8–10] It was seen in these studies that the calculated rectal dose was different in the two methods (rectal wire and ICRU-38). On an average the rectum receives a 15% higher dose as compared to the dose calculated by the intrarectal wire method. Our study also confirms these findings. The most significant difference was seen in the R1 and R5 pair, i.e., at the level of the cervical os or the lowest level of the intrauterine source. At the other points also (R2 and R6, R3 and R7, R4 and R8) a difference was present between the ICRU-38 calculation and the intrarectal wire method, but it was small. The average dose at R1 was 5% higher than that at R5 (60.57% vs. 55.57%), which was very significant when the statistical tests were applied (p value = 0.002). It clearly shows that at this point the dose estimation by the usual intrarectal method, underestimates the actual dose. The lower dose in the intrarectal method arises due to the fact that these rectal wire markers are inserted randomly in the rectal lumen and usually do not fill the whole rectum due to their smaller diameter. Furthermore, variation in diameter of the rectal wire can change the position of the rectal points in this method. Using a catheter with diameter smaller than the rectal lumen may lead to wrong marking of the rectal points, if the catheter is not in close approximation with the anterior rectal wall. Similarly, if a larger diameter is used it may actually push the anterior wall of the rectum further anteriorly, again leading to wrong marking of the rectal point. Thus positions of these rectal markers are variable, thereby leading to false calculation of the rectal dose. This will lead to a lot of variation in reporting from center to center. Therefore, specific rectal points determined in this manner cannot represent the true rectal wall dose.

The precise location of the rectum is possible on CT slice by CT-based dosimetry.[7,11] It is not possible to introduce image-based brachytherapy (CT based) in daily clinical practice everywhere as it is expensive, time consuming, and not available at most centers. Thus, for centers with limited resources, it is best to stick to the ICRU guidelines, which require just orthogonal films.

## Conclusion

By comparing the ICRU-38 recommended rectal point and the intrarectal catheter derived rectal point, it can be seen that the actual dose received is high, as seen in the ICRU-38 method. The intrarectal catheter falsely shows a lower value at the rectal point. Thus, till modern image-based brachytherapy replaces the conventional system, one should stick to the ICRU guidelines for dose estimation.
